# Polarization-Multiplexed Binocular Metasurface-Empowered Monolithic Ranging and Dual-Mode Imaging System

**DOI:** 10.1021/acsphotonics.6c00927

**Published:** 2026-10-06

**Authors:** Zixuan Wang, Chuang Sun, Yixuan Zhao, Kian Shen Kiang, Jun-Yu Ou, Jize Yan

**Affiliations:** † School of Electronics and Computer Science, 7423 University of Southampton, Southampton SO17 1BJ, U.K.; ‡ School of Physics and Astronomy, University of Southampton, Southampton SO17 1BJ, U.K.

**Keywords:** Metasurface, Polarization multiplexing, Dual-mode imaging, Edge-Enhancement Imaging, Depth ranging

## Abstract

Metasurfaces provide a compact platform for multifunctional optical systems. Their applications have expanded to three-dimensional imaging and depth sensing. Passive binocular imaging determines depth from disparity between laterally separated views, and its metasurface-based implementations have been widely explored for compact depth sensing. However, passive binocular ranging remains limited by the finite depth of focus in conventional Gaussian imaging systems. Away from the focal plane, defocus reduces spatial intensity gradients and degrades feature localization accuracy. Here, we report a polarization-multiplexed binocular metasurface system that integrates dual-mode imaging and passive depth sensing within a single architecture. The system employs two identical metalenses separated by a fixed baseline. Each metalens supports polarization-dependent Gaussian and orbital-angular-momentum (OAM) focusing modes to acquire bright-field and phase-contrast images. The complementary information from the two imaging modes enables edge-enhanced images with improved feature visibility even under defocus, supporting feature localization across varying axial positions. Experiments demonstrate passive depth reconstruction with an average relative error of 4.9% over a working distance from 15 mm to 200 mm. This system enables compact integrated imaging and passive depth sensing using a single metasurface.

## Graphical abstract

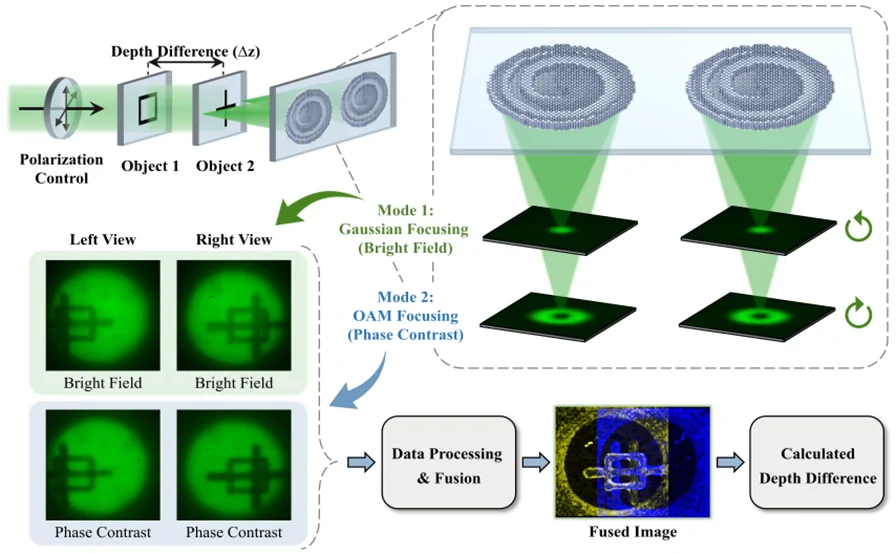

## 1 Introduction

Passive binocular stereoscopic ranging determines object depth from disparity between laterally separated views.^1–5^ Unlike conventional depth-sensing techniques, such as time-of-flight (ToF)^6,7^ and structured illumination,^8–11^ which encode depth through temporal delay or projected patterns, passive binocular ranging relies on geometric triangulation. This approach eliminates the need for active illumination and complex detection schemes, enabling compact and energy-efficient depth sensing. However, accurate disparity estimation depends on reliable feature localization across the two imaging channels. In conventional Gaussian imaging systems, axial defocus reduces spatial intensity gradients and degrades localization accuracy, leading to errors in depth estimation.

Metasurfaces provide subwavelength control of optical phase, amplitude and polarization within compact planar architectures.^12–16^ In addition to conventional imaging, metasurfaces enable system-level control of the optical phase profile, which supports the design of optical field distributions.^17^ In particular, optical fields carrying orbital angular momentum (OAM) can be generated by spiral phase modulation.^14,18–21^ These fields produce vortex focal distributions that suppress uniform intensity components while enhancing responses to spatial gradients. This property enables phase-contrast imaging. Importantly, gradient information is preserved during axial propagation, allowing feature localization to remain reliable under defocus.

Metasurface-based approaches have been explored for three-dimensional imaging and depth sensing. Monocular methods encode depth into engineered point-spread functions, chromatic dispersion, or phase-mask-induced patterns.^8,22,23^ In such systems, depth retrieval relies on computational inversion or model-based reconstruction. Binocular metasurface systems have also been demonstrated.^1–5^ However, existing configurations employ conventional focusing in each channel. Approaches based on optical field design to preserve spatial gradients under defocus remain underexplored.

Here, we present a polarization-multiplexed binocular metasurface imaging system designed for passive stereoscopic ranging. The system consists of two metalenses separated by a fixed baseline, each supporting polarization-controlled switching between Gaussian focusing for bright-field imaging and OAM focusing for phase-contrast imaging. The two imaging modes provide complementary responses to spatial gradients, enabling edge-enhanced feature extraction. The resulting feature maps exhibit increased robustness under defocused conditions. The relative disparity between the two channels encodes object depth. By combining polarization-controlled focal-mode switching with binocular metasurface, the system achieves simultaneous imaging and depth sensing without additional optical elements. Experimental results demonstrate an average relative depth reconstruction error of 4.9% over a working distance from 15 mm to 200 mm. The approach offers a compact platform for integrated imaging and passive stereoscopic ranging.

## 2 Results and Discussion

Figure 1 illustrates the working principle of the binocular metasurface imaging system. The binocular system consists of two metalenses with identical optical designs and a fixed center-to-center baseline of 5 mm. Each metalens supports two polarization-dependent focusing modes. Under left-handed circularly polarized (LCP) illumination, a Gaussian focal spot is generated for bright-field imaging. Under right-handed circularly polarized (RCP) illumination, a vortex focal field with an annular intensity distribution is produced for phase-contrast imaging. The two laterally displaced imaging channels introduce measurable disparity for depth estimation. The dual-mode operation further provides complementary responses to spatial gradients, which are used for feature extraction.

**Figure 1:**
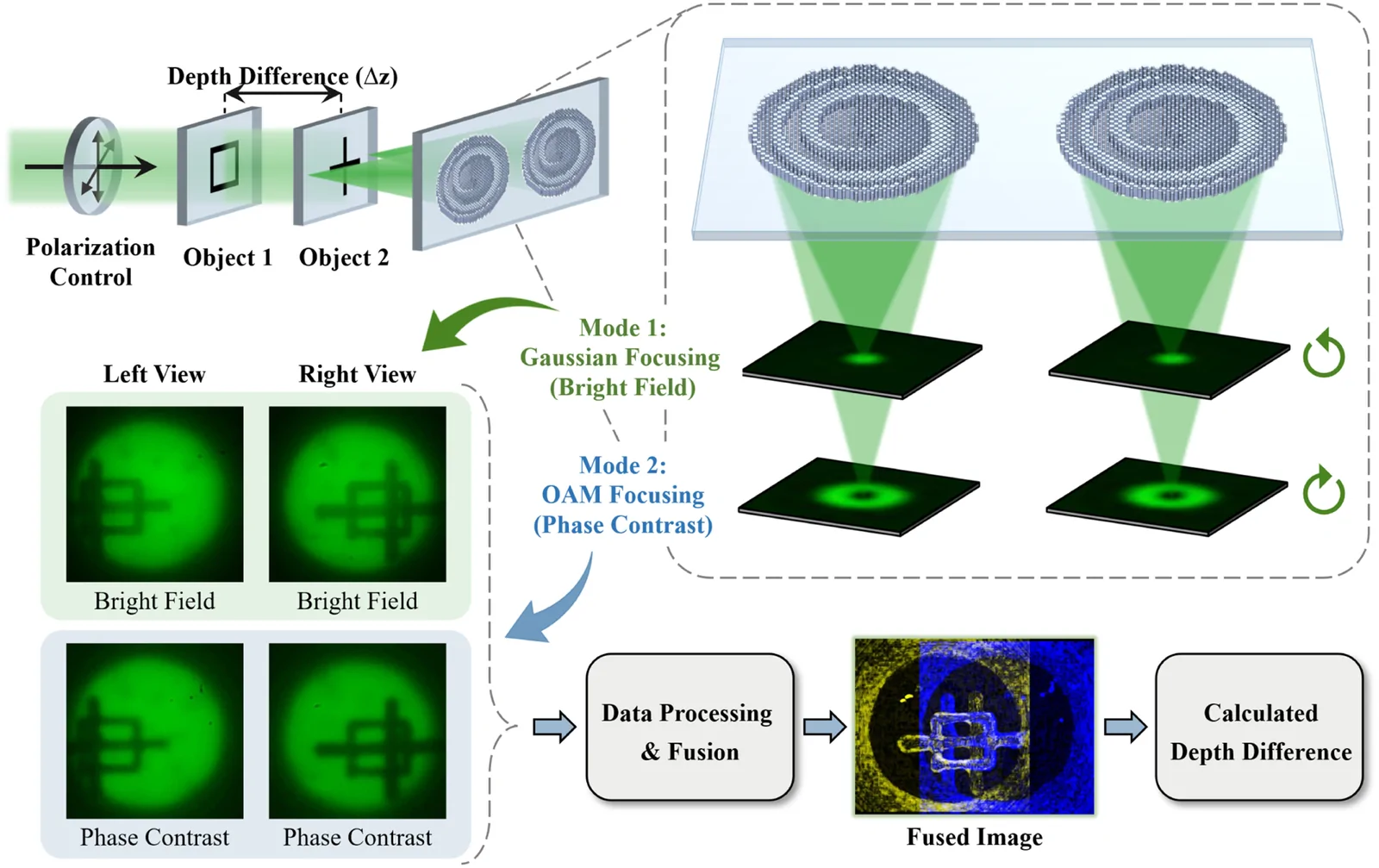
Conceptual schematic of the polarization-multiplexed binocular metasurface system for simultaneous edge-enhanced imaging and ranging. (Top) Experimental configuration, where polarization-controlled illumination is incident on objects with different axial positions and collected by a single-substrate binocular metasurface. (Middle) Illustration of the two polarization-dependent operation modes: Mode 1 generates Gaussian focal spots for bright-field imaging, while Mode 2 produces OAM focal spots for phase-contrast imaging. (Bottom) Representative raw images acquired from the left and right viewpoints and the corresponding data processing workflow.

All metasurfaces are designed to operate at a wavelength of 520 nm. A spin-multiplexed meta-atom library reported in our previous work is employed to achieve high transmission efficiency and full 2π phase coverage.^24^ The meta-atoms have a height H = 800 nm and a period P = 350 nm. The library provides transmission coefficients *t_x_* and *t_y_* exceeding 90% for both orthogonal polarization states.

Based on this meta-atom library, the phase profiles of the metalens are designed using a spin-multiplexing strategy. Under LCP illumination, the metalens applies a hyperbolic phase profile designed for a focal length *f*_1_ = 20 mm. Under RCP illumination, the metalens applies a composite phase profile that includes a hyperbolic phase with focal length *f*_2_ = 24 mm and a spiral phase term with topological charge *l* = 1, generating a vortex focal field. The phase profiles are given by

1 $$\psi_{L}=\frac{2\pi }{\lambda }\left(f_{1}-\sqrt{{f_{1}}^{2}+r^{2}}\right)$$

2 $$\psi_{R}=\frac{2\pi }{\lambda }\left(f_{2}-\sqrt{{f_{2}}^{2}+r^{2}}\right)+l\phi$$

where (*r*, *ϕ*) are the polar coordinates of the aperture. Both metalenses use the same design to ensure consistent optical behavior in the left and right imaging channels. Switching between bright-field and phase-contrast modes is achieved solely by controlling the incident polarization state, without modifying the optical system.

Figure 2 a and 2b show an optical photograph and a scanning electron microscope (SEM) image of the fabricated metasurface, respectively. The focusing performance was characterized under controlled polarization states using the setup shown in Figure 2(c). The experimental setup consisted of a 520 nm laser, a linear polarizer, a half-wave plate (HWP), a quarter-wave plate (QWP), steering mirrors, the metasurface, and an imaging microscope. The polarizer defined the initial polarization state, while the HWP–QWP pair generated the desired incident circular polarization. The transmitted light was then collected by a 10× objective lens (NA = 0.3) and relayed by a 200 mm tube lens onto a CMOS camera (DCC1545M, Thorlabs). The effective magnification was calibrated to be 7.9× using a USAF 1951 resolution target (R1DS1N, Thorlabs).

**Figure 2:**
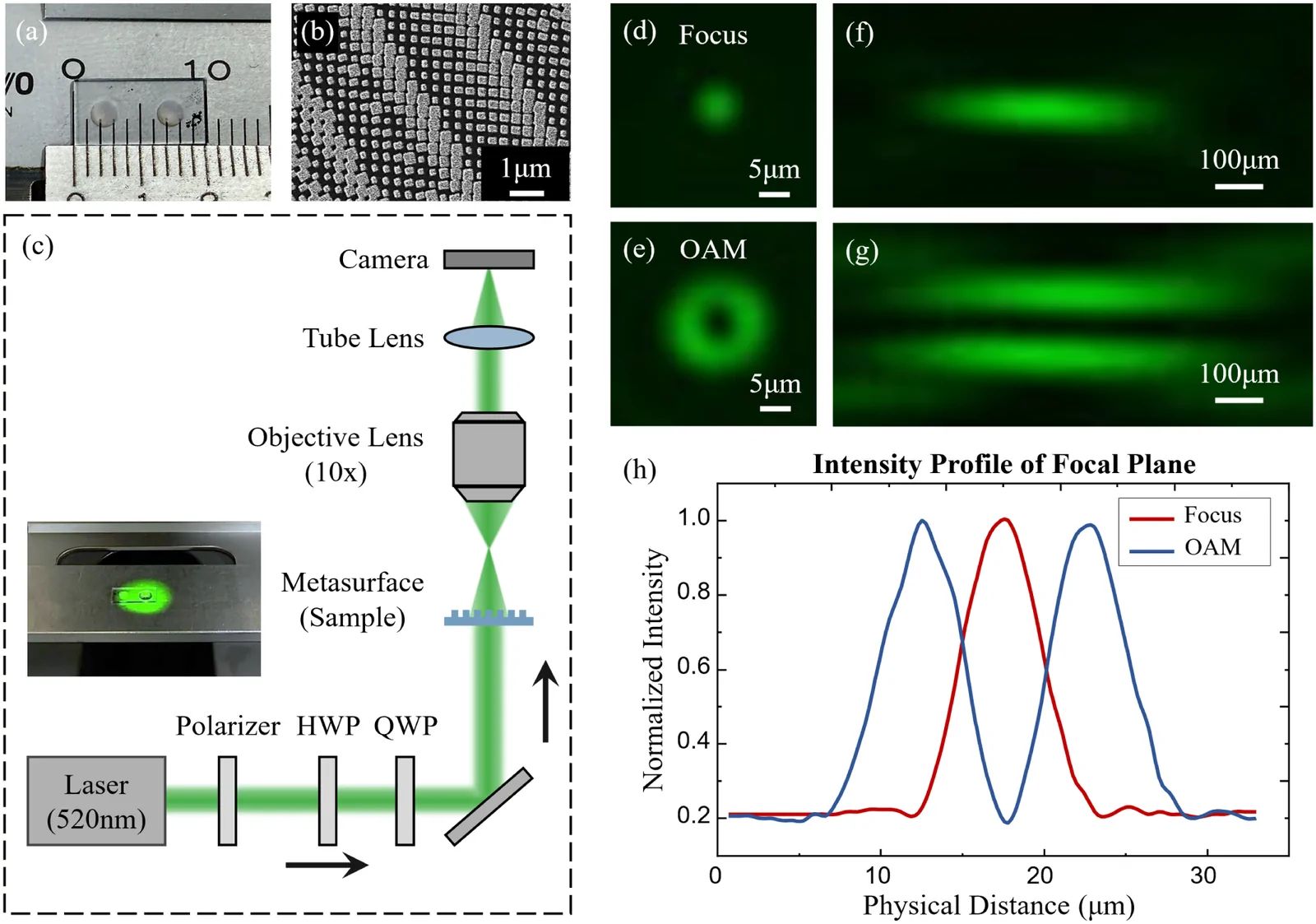
Optical characterization of the polarization-multiplexed metasurface. (a) Picture and (b) SEM image of the metasurface. (c) Experimental setup for focal characterization. (d, e) Measured focal-plane intensity distributions under (d) LCP illumination corresponding to the focusing mode and (e) RCP illumination corresponding to the OAM mode. (f, g) Axial intensity distributions obtained by scanning the imaging plane around the focal position for the (f) Gaussian focusing mode and the (g) OAM mode. (h) Normalized transverse intensity profiles extracted from the focal-plane images in (d) and (e) for full width at half-maximum (fwhm) analysis.

Focusing measurements were first performed under LCP illumination, corresponding to the Gaussian focusing mode with a designed focal length of 20 mm. By translating the microscope imaging plane along the optical axis, a well-defined focal spot was observed at a distance of 19.84 mm from the metasurface, as shown in Figure 2d. The incident polarization was then switched to RCP to characterize the OAM focusing mode with a designed focal length of 24 mm. An annular intensity distribution was observed during the axial scan, with maximum ring visibility at an axial distance of 23.8 mm, as shown in Figure 2e. Both values are in good agreement with the corresponding design parameters. The axial intensity profiles were further measured by scanning the imaging plane over a 1 mm range with a step size of 20 μm, as shown in Figure 2f and 2g. The depth of focus (DOF), defined as the full width at half-maximum (fwhm) of the axial response, was measured to be approximately 550 μm for the Gaussian mode and 900 μm for the OAM mode.

Figure 2h shows the normalized intensity profiles extracted at the focal plane for the two modes. For the Gaussian mode, the fwhm was directly measured to be approximately 5.4 μm. For the OAM mode, the measured fwhm values were approximately 5.54 and 5.26 μm for the two peaks, with an average fwhm of approximately 5.4 μm. In addition, the peak-to-peak (PTP) separation of the annular focal spot in the OAM mode was measured to be 9.9 μm, which agrees well with the expected transverse intensity distribution of a vortex beam with a topological charge of *l* = 1. These results confirm polarization-controlled switching between Gaussian and OAM focusing modes while maintaining near-diffraction-limited focusing performance in both cases.

## 3 Experimental Section

### 3.1 Dual-Mode Imaging and Edge Enhancement

To evaluate the imaging performance, an infinity-corrected imaging system was constructed in which the metalens simultaneously serves as the objective lens (Figure 3a). Illumination was provided by a 520 nm LED source, and the incident polarization state was controlled using a linear polarizer, a half-wave plate, and a quarter-wave plate. The polarized light was then focused onto a USAF 1951 resolution target (R1DS1N, Thorlabs) using a condenser lens. Then the light was collected by the metalens and relayed through a 100 mm tube lens (AC254–100-C, Thorlabs) onto a scientific CMOS camera (DCC1545M, Thorlabs). This configuration resulted in theoretical magnifications of 5× and 4.25× for the two imaging modes.

**Figure 3:**
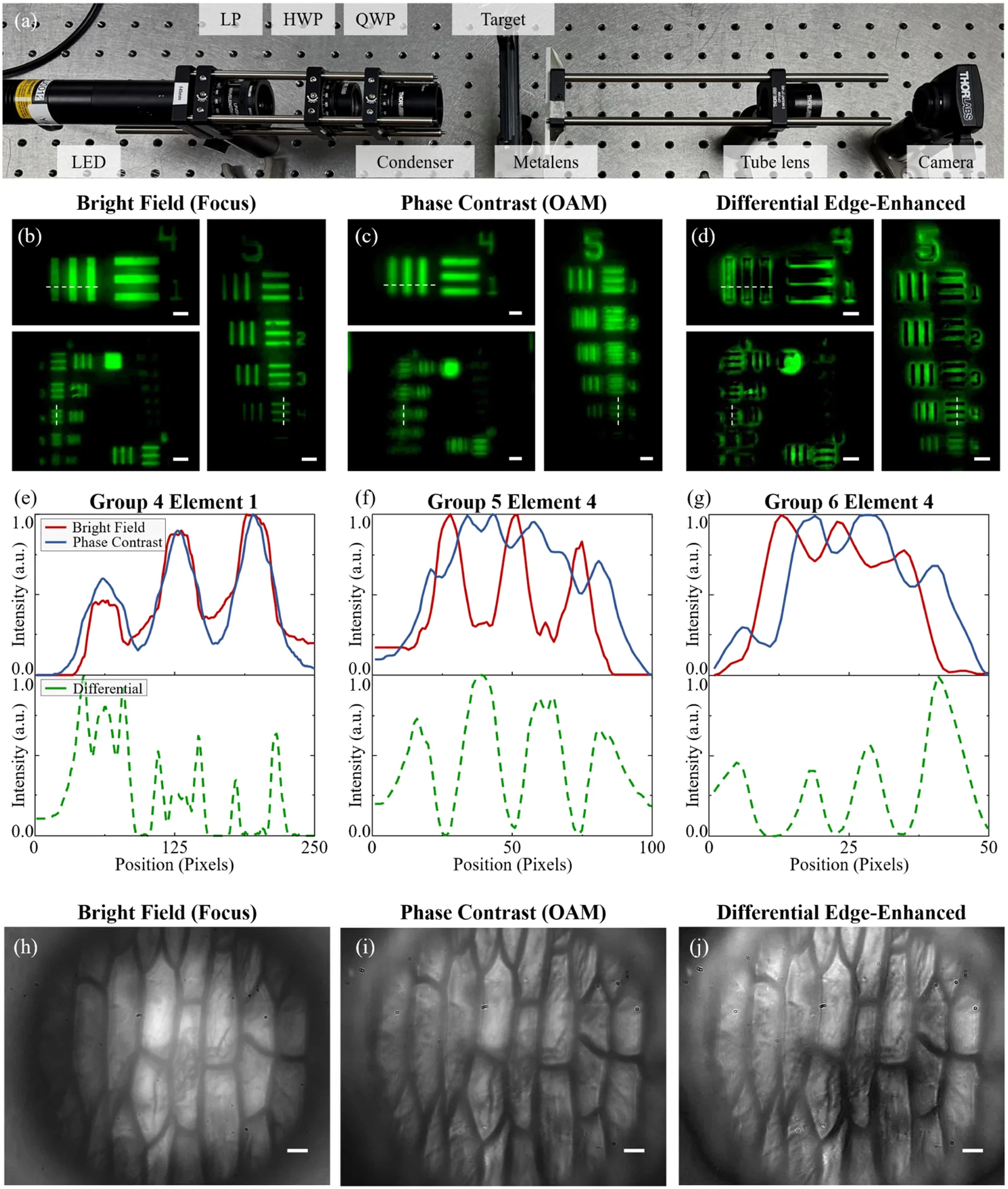
Dual-mode imaging of resolution targets and biological samples using the metasurface-based microscope. (a) Schematic of the imaging setup. (b, c) Images of a USAF 1951 resolution target acquired under (b) bright-field imaging and (c) phase-contrast imaging, respectively. (d) Differential image obtained by single-step subtraction of the phase-contrast image and the corresponding bright-field image. (e–g) Normalized intensity profiles extracted along the white dashed lines in (b–d) for (e) Group 4 Element 1, (f) Group 5 Element 4, and (g) Group 6 Element 4. The red solid line denotes the bright-field image, the blue solid line denotes the phase-contrast image, and the green dashed line denotes the differential image. (h–j) Images of onion epidermal cells acquired under (h) bright-field imaging, (i) phase-contrast imaging, and (j) the corresponding differential image. Scale bars: 50 μm.

Imaging tests were first conducted under LCP illumination corresponding to the bright-field imaging mode, and were subsequently repeated under RCP illumination to realize phase-contrast imaging. Figure 3b and 3c show the images obtained under bright-field (LCP) and phase-contrast (RCP) modes, respectively. Due to the different focal lengths, the measured magnifications are 5.1× and 4.3×, in agreement with the design. To enable direct comparison and differential processing, the phase-contrast image was rescaled by a factor of 1.2 to match the effective magnification of the bright-field image. A differential image was then obtained by subtracting the two aligned images, as shown in Figure 3d.

The bright-field image (Figure 3b) exhibits intensity maxima within the transparent regions of the patterns, as expected for conventional imaging. In contrast, the phase-contrast image (Figure 3c) shows enhanced intensity near structural boundaries and reduced intensity in uniform regions. This difference originates from the phase-contrast response of the metalens, which suppresses spatially uniform components while emphasizing intensity variations associated with structural discontinuities. This effect becomes more pronounced for higher spatial frequencies. In the USAF target, higher group numbers correspond to smaller feature sizes and higher spatial frequencies. For these finer structures, the phase-contrast image shows stronger localization of intensity near the edges. Although the visual contrast varies with feature size, the edge-associated information remains encoded across spatial scales. After differential processing, uniform background components are largely removed, while edge-associated signals are enhanced, as shown in Figure 3d.

The contrast behavior is further analyzed using normalized intensity profiles extracted from representative resolution elements (Group 4 Element 1, Group 5 Element 4, and Group 6 Element 4), as shown in Figure 3(e–g). The bright-field profiles (red solid lines) exhibit intensity maxima at the centers of the transparent bars, while the phase-contrast profiles (blue solid lines) show reduced intensity at the centers and enhanced responses near the edges. After differential processing, the resulting profiles (green dashed lines) exhibit narrow peaks localized at the boundaries. At the same time, the peak-to-valley contrast is significantly increased, indicating that the differential operation effectively enhances the edge signals while suppressing the background intensity.

To reduce interference artifacts associated with coherent illumination, all measurements were performed using a partially coherent LED source. The use of an LED source also improves compatibility with biological imaging. For comparison, imaging experiments under laser illumination are presented in Supporting Information S2. Under coherent illumination, edge-enhanced features can be directly observed in the vortex-assisted mode without differential processing. The system resolves Group 6 Element 4 (5.5 μm) in both imaging modes, close to the diffraction-limitations.

To further evaluate performance on biological samples, onion epidermal cells were imaged under LED illumination. Figure 3(h–j) show the bright-field, phase-contrast, and differential images, respectively. The bright-field image (Figure 3h) captures the overall morphology, while the phase-contrast image (Figure 3i) highlights intensity variations associated with cell boundaries. After rescaling the phase-contrast image to match the effective magnification of the bright-field image, differential processing was applied to obtain the edge-enhanced image shown in Figure 3j. The resulting differential image provides enhanced visualization of cell boundaries and local surface features compared with either individual imaging mode.

### 3.2 Passive Binocular Depth Reconstruction

To evaluate the depth-ranging capability of the proposed metasurface system, 3D ranging experiments were performed using a binocular configuration composed of two laterally separated metalenses. As illustrated in Figure 4a, the system consists of LED illumination, polarization-control system, a condenser lens, the test object, a binocular metasurface, a relay imaging system, and a camera. The relay system consists of two lenses with focal lengths of 30 mm and 150 mm, and the lenses and camera are mounted on a rigid cage system to ensure stable alignment and focus. In this configuration, the object is illuminated by the condenser lens and directly imaged by the two metalenses. The resulting images are relayed and magnified before being recorded by the camera. This setup differs from the resolution characterization system in that stereo imaging is performed using two laterally separated imaging channels.

**Figure 4:**
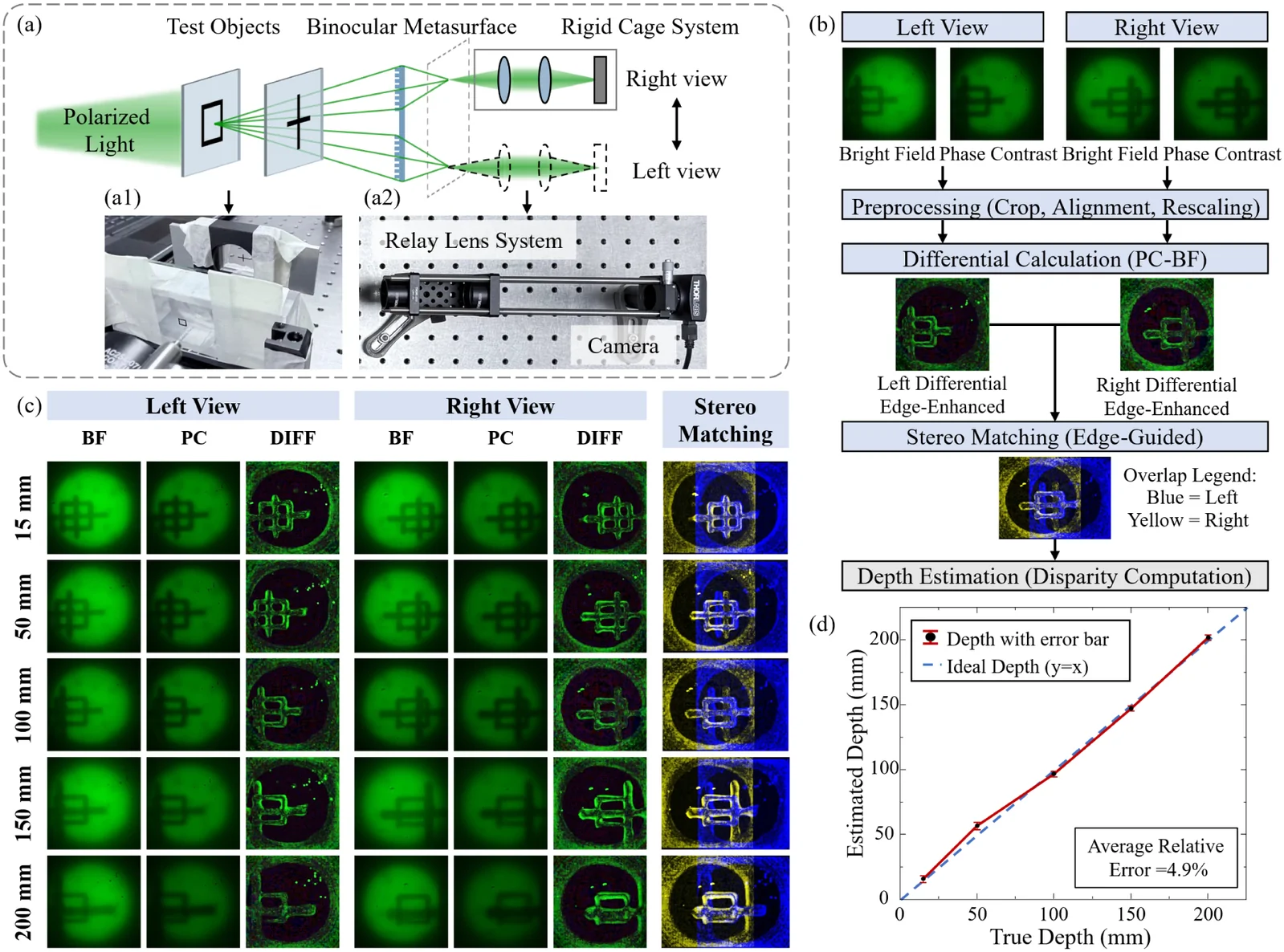
Dual-mode binocular imaging and depth estimation using a polarization-controlled metasurface. (a) Schematic illustration of the binocular imaging setup. (b) Data processing workflow for depth estimation. (c) Representative imaging results obtained over a depth range of 15–200 mm in bright-field (BF), phase-contrast (PC), and differential (DIFF) modes. (d) Quantitative depth reconstruction results compared with ground-truth values.

The test object consisted of two patterns drawn manually on two separate glass slides, as shown in Figure 4a1. A square pattern was fixed at a distance of 450 mm from the metasurface and served as a reference object. A cross-shaped pattern was placed closer to the metasurface and served as the target. The axial position of the target was varied from 15 mm to 200 mm relative to the reference, with five discrete measurement positions, and images were recorded at each position. The measurement was terminated when the vertical arm of the cross pattern moved completely outside the field of view. For each measurement, images were acquired under both polarization-controlled imaging modes by sequentially adjusting the incident polarization (Figure 4a2). Due to the limited field of view, the left and right views were recorded sequentially by laterally translating the relay imaging system and camera. This ensured consistent imaging conditions for both viewpoints. The subsequent data processing workflow is shown in Figure 4b, including preprocessing, differential edge extraction, stereo matching, and disparity-based depth estimation.

The experimental results across the measured depth range of z = 15 mm to 200 mm are shown in Figure 4c. The columns display the raw bright-field (BF) intensity distributions and the corresponding upscaled phase-contrast (PC) features acquired from the binocular system. As the cross-shaped target moves closer to the metasurface relative to the square-patterned reference, the images exhibit increased magnification and defocus-induced blur. The third column (DIFF) shows the result of differential processing between the two imaging modes. Defocus degrades the bright-field and phase-contrast images, whereas the differential images preserve well-defined object boundaries and edge structures. Compared with the raw bright-field images, they provide more precise edge localization and improved visibility of structural features. The fourth column in Figure 4c visualizes the stereo matching results, where the edge features extracted from the two views are overlaid in blue and yellow colors, respectively. The lateral displacement between the matched features represents the disparity, which encodes the relative depth between the target and the reference. Larger disparities correspond to larger depth differences.

To quantitatively validate the depth-ranging performance, the estimated depth differences were compared with the ground-truth positions. For each position, multiple feature correspondences were extracted, and the disparity was averaged to obtain a single depth estimate. As shown in Figure 4d, the measured depth differences follow a linear relationship with the ground truth. The fitted curve closely matches the ideal response (y = x), confirming accurate depth reconstruction. The vertical error bars denote one standard deviation of the estimated depth differences, calculated from multiple spatial correspondences within each depth plane. Across the full depth range, the standard deviation remains low (approximately 1.8–2.8 mm), indicating consistent disparity extraction. The relative error was calculated as the absolute difference between the estimated and ground-truth depths normalized by the ground-truth value. The average relative error across all measurements is 4.9%, remaining below 5% over the entire depth range. Higher accuracy is observed at larger depth separations, with a relative error as low as 0.88% at a depth difference of 200 mm. These quantitative results demonstrate that the polarization-controlled dual-mode metasurface system enables accurate and stable passive depth ranging by combining bright-field structural information with phase-contrast edge-enhanced feature extraction.

## 4 Conclusion

In conclusion, we have demonstrated a metasurface-based binocular imaging system that integrates dual-mode imaging and passive stereoscopic depth sensing within a compact architecture. The spin-multiplexed metalenses enable polarization-controlled switching between Gaussian and OAM focusing modes, providing complementary structural and edge information to be extracted from a single optical element. When implemented in a binocular configuration, this dual-mode operation enables reliable disparity extraction without active illumination or additional optical components. Experimental results demonstrate diffraction-limited focusing in both imaging modes. Edge enhancement under defocus enables depth reconstruction with an average error below 5%. Biological imaging further confirms the applicability to weakly scattering samples. The demonstrated approach offers a practical pathway toward multifunctional flat-optics platforms and may benefit applications in portable 3D sensing, machine vision, robotics, and biomedical microscopy. Future developments may extend this concept to broadband operation, dynamic phase modulation, and wafer-scale integration for scalable depth-sensing systems.

## Supplementary Material



## Notes

Details of the metasurface fabrication process and structural characterization (Figure S1), as well as additional imaging results under coherent illumination (Figure S2) (PDF)
