# Supplementary material for: Polarization-Multiplexed Binocular Metasurface-Empowered Monolithic Ranging and Dual-Mode Imaging System
Source: ACS Photonics. 2026 Oct 6;13(20):6060–6. doi: 10.1021/acsphotonics.6c00927 (PMC13693497; doi:10.1021/acsphotonics.6c00927)
Supplement: Supplementary file 1 [file ph6c00927_si_001.pdf]

Supporting Information

Polarization-Multiplexed Binocular Metasurface Empowered Monolithically Ranging and Dual-Mode Imaging System

Zixuan Wang[1], *Chuang Sun*[1], *Yixuan Zhao*[1], *Kian Shen Kiang*[1], *Jun-Yu Ou*[2], *and Jize Yan*[1,*]

[1] School of Electronics and Computer Science, University of Southampton, Southampton, SO17 1BJ, United Kingdom

[2] School of Physics and Astronomy, University of Southampton, Southampton, SO17 1BJ, United Kingdom

*E-mail: J.Yan@southampton.ac.uk

1. Fabrication of metasurface

The fabrication process is illustrated in Fig. S1 and was carried out at the Southampton Nanofabrication Centre using a CMOS-compatible process flow. The metasurface was fabricated on a 0.5 mm thick double-side-polished silicon dioxide wafer. An 800 nm thick SiN

layer was deposited by plasma-enhanced chemical vapor deposition (PECVD). Then, a 300 nm thick ZEP520A electron-beam resist and an Espacer conductive layer were spin-coated and patterned by electron beam lithography (EBL) from the design mask to the resist layer. After development, a 30 nm thick chromium (Cr) layer was deposited by evaporation to serve as a hard mask. Reactive ion etching (RIE) was then used to transfer the pattern into the SiN layer. Finally, the Cr hard mask was removed by wet etching using a chromium etchant.

The optical microscope image in Fig. S1(b) shows the overall spiral phase pattern. SEM images in Fig. S1(c,d) show the central and edge regions, respectively. The edge region contains rapidly varying features for evaluating pattern fidelity across different structure sizes.

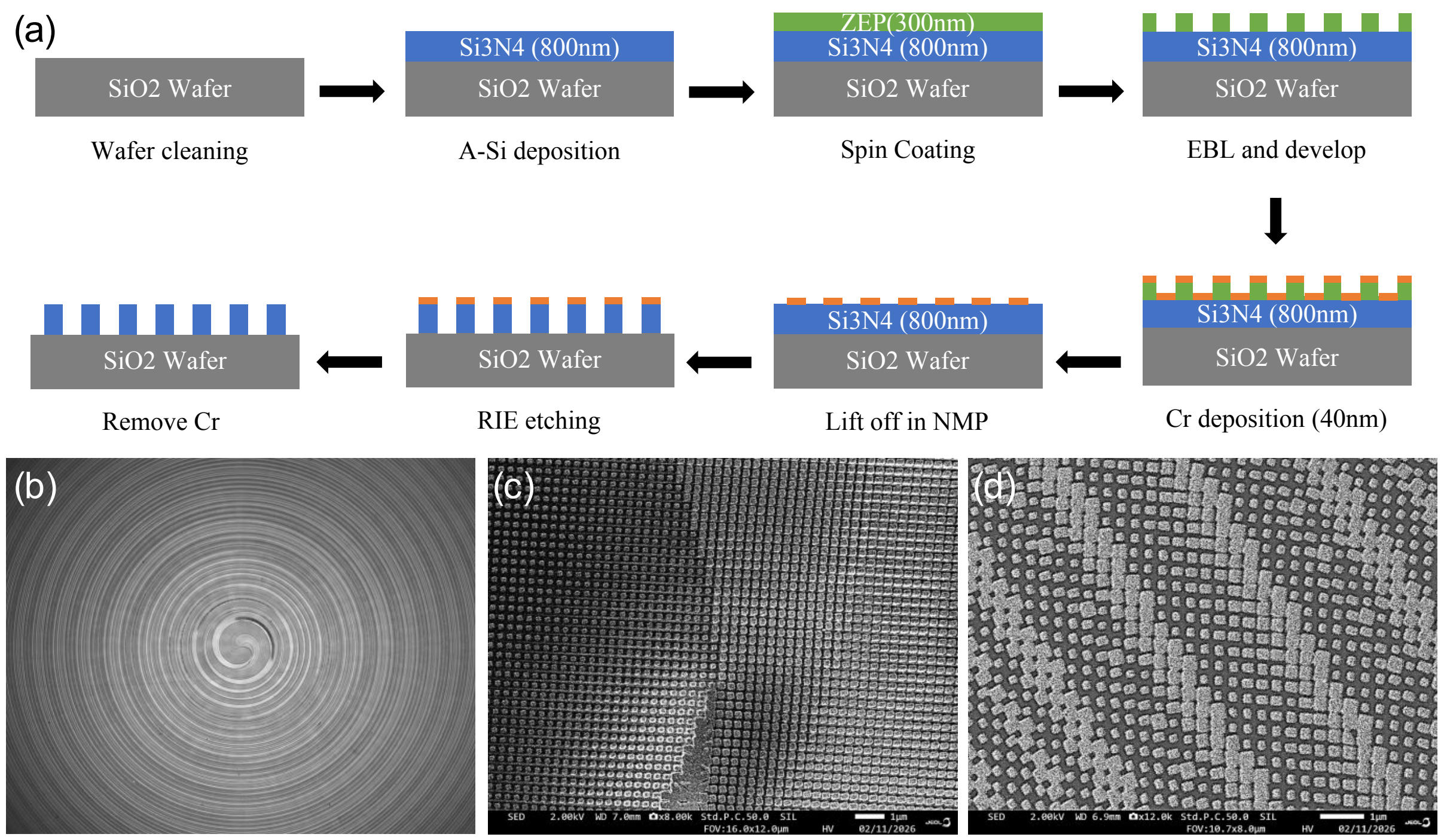

Figure S1. Fabrication process and structural characterization of the metasurface.

(a) Fabrication process flow. (b) Optical microscope image of the fabricated metasurface. (c) SEM image of the central region of the metasurface. (d) SEM image of the edge region.

2. Imaging Results Under Coherent Illumination

To further evaluate the imaging behavior of the metasurface system under coherent illumination, imaging experiments were performed using a 520 nm laser source. The same optical configuration as in the main text was used. Figure S2 presents representative imaging results. The bright-field images exhibit conventional intensity distributions, while the phase-contrast images show enhanced responses at feature boundaries. The system resolves Group 6 elements in both imaging modes, consistent with the focusing performance characterized in the main text. Under coherent illumination, the metasurface directly produces edge-enhanced images without requiring differential processing. The partially coherent LED illumination used in the main text provides a more practical configuration for general imaging applications.

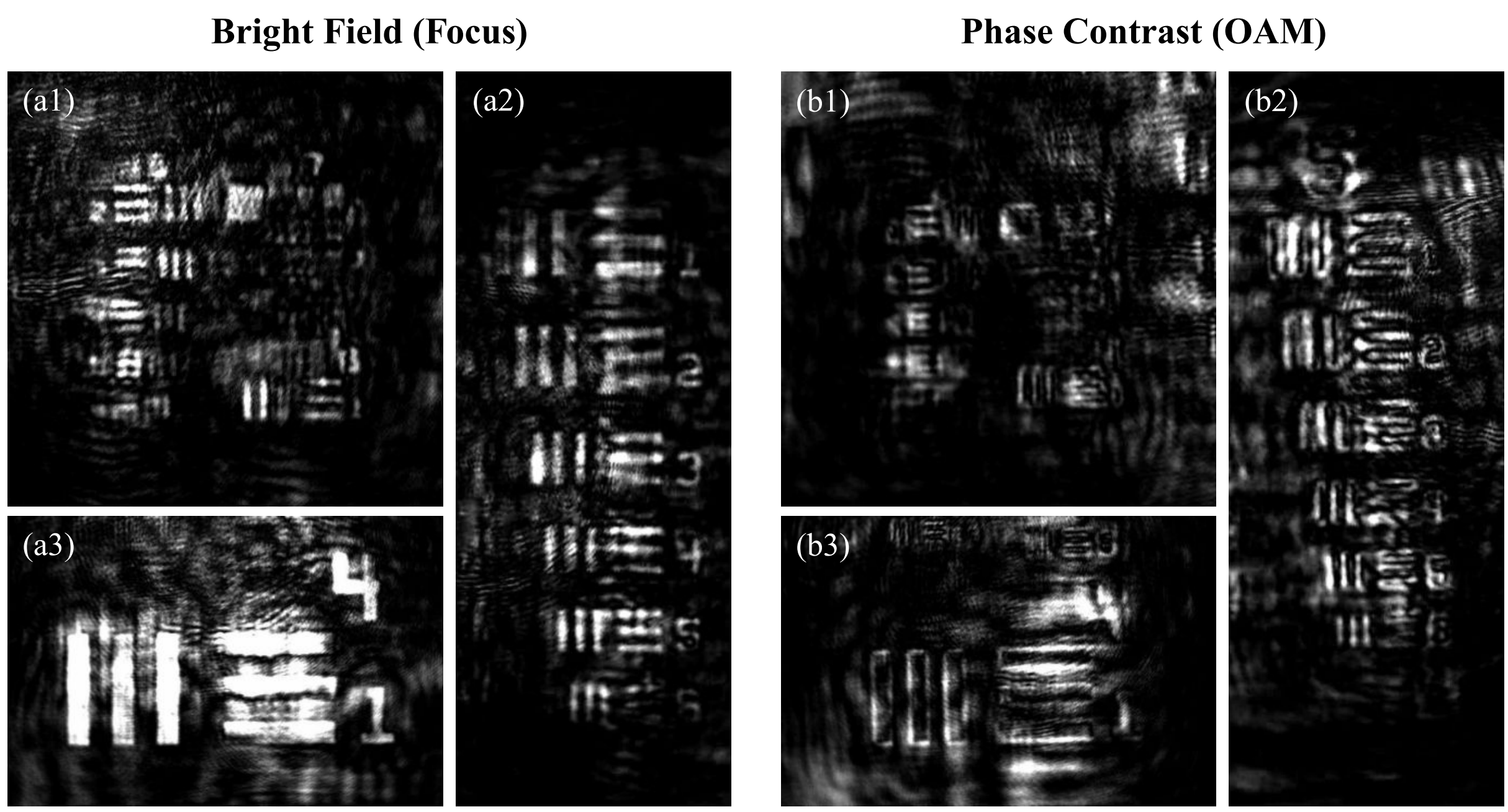

Figure S2. Imaging results of the metasurface-based microscope under coherent illumination. (a,b) Images of a USAF 1951 resolution target acquired under (a) bright field imaging and (b)

phase contrast imaging, respectively. (a1–a3) Bright field images corresponding to Group 6, Group 5, and Group 4, respectively. (b1–b3) Phase contrast images corresponding to Group 6, Group 5, and Group 4, respectively.
